# Pilot study of humanized glypican-3-targeted zirconium-89 immuno-positron emission tomography for hepatocellular carcinoma

**DOI:** 10.21203/rs.3.rs-4456645/v1

**Published:** 2024-06-27

**Authors:** Lindsay K. Dickerson, Adrienne L. Lehnert, Donald K. Hamlin, Kevin P. Labadie, Kristin E. Goodsell, Yongjun Liu, Yawen Li, D. Scott Wilbur, Robert Miyaoka, James O. Park

**Affiliations:** University of Washington; University of Washington; University of Washington; University of Washington; University of Washington; University of Washington; University of Washington; University of Washington; University of Washington; University of Washington

**Keywords:** hepatocellular carcinoma, glypican-3 (GPC3), Zirconium-89, immuno-positron emission tomography (immunoPET), theranostics

## Abstract

**Purpose:**

Glypican-3 (GPC3)-targeted radioisotope immuno-positron emission tomography (immunoPET) may lead to earlier and more accurate diagnosis of hepatocellular carcinoma (HCC), thus facilitating curative treatment, decreasing early recurrence, and enhancing patient survival. We previously demonstrated reliable HCC detection using a zirconium-89-labeled murine anti-GPC3 antibody (89Zr-αGPC3M) for immunoPET. This study evaluated the efficacy of the humanized antibody successor (αGPC3H) to further clinical translation of a GPC3-based theranostic for HCC.

**Methods:**

In vitro αGPC3 binding to HepG2 cells was assessed by flow cytometry. In vivo 89Zr-αGPC3H and 89Zr-αGPC3M tumor uptake was evaluated by PET/CT and biodistribution studies in an orthotopic xenograft mouse model of HCC.

**Results:**

αGPC3H maintained binding to GPC3 in vitro and 89Zr-αGPC3H immunoPET identified liver tumors in vivo. PET/CT and biodistribution analyses demonstrated high 89Zr-αGPC3H tumor uptake and tumor-to-liver ratios, with no difference between groups.

**Conclusion:**

Humanized αGPC3 successfully targeted GPC3 in vitro and in vivo. 89Zr-αGPC3H immunoPET had comparable tumor detection to 89Zr-αGPC3M, with highly specific tumor uptake, making it a promising strategy to improve HCC detection.

## Introduction

Hepatocellular carcinoma (HCC) is increasing in incidence worldwide and has become the fastest growing cause of cancer death in the United States, with a median survival of less than one year [1–3]. In order to improve survival with current treatments, HCC must be detected early when it is amenable to surgical resection or transplantation [3.^4^]. However, multiphase, computed tomography (CT) or magnetic resonance imaging frequently misses lesions less than 1 cm, resulting in diagnostic uncertainty, delayed diagnosis, and early recurrence following resection [5, 7]. Innovative technology capable of detecting HCC with enhanced sensitivity and specificity is therefore imperative and pressing.

Radioisotope theranostics, including immuno-positron emission tomography (immunoPET) and radioimmunotherapy (RIT), is an emerging field with the potential to transform HCC diagnosis and therapy [8]. While yttrium-90 microspheres and iodine-131-labeled lipiodol and metuximab are used in radioembolization therapy, there are currently no FDA approved theranostics for HCC. However, glypican-3 (GPC3)-targeted radioisotopes have shown promise in preclinical and early clinical studies [6, 9–20]. GPC3 is a cell surface antigen expressed on up to 80% of HCCs but absent in liver parenchyma and benign lesions, making it an accessible and specific target for a theranostic approach [14, 15, 21]. GPC3-based imaging has the potential to facilitate earlier, definitive HCC diagnosis and subsequent RIT, thus improving patient survival [16].

Our group previously demonstrated that immunoPET using zirconium-89 (^89^Zr)-labeled murine antibody targeting GPC3 (^89^Zr-αGPC3_M_) reliably identified small HCCs in mice [6, 10, 11]. Natarajan et al. described the use of ^89^Zr-labeled humanized αGPC3 for HCC detection in a patient-derived xenograft model [16]. We built on this important work by humanizing our radioimmunoconjugate (αGPC3_H_) and performing *in vitro* and novel *in vivo* comparisons to its murine predecessor. Here, we report that ^89^Zr-αGPC3_H_ targets GPC3 comparably to ^89^Zr-αGPC3_M_, resulting in highly specific tumor uptake and successful HCC detection.

## Materials and Methods

Creative Biolabs, Inc. (Shirley, NY) constructed αGPC3_H_ by engrafting of the parental murine antibody’s complementarity-determining region (CDR). Flow cytometry was used to evaluate *in vitro* binding of αGPC3_M_, a chimeric intermediary (αGPC3_C_), αGPC3_H_, and αGPC3-deferoxamine (DFO) to HepG2 cells. Orthotopic xenograft models of HCC were generated as previously described in athymic nude mice (Jackson Laboratories) [10–12, 22]. Two weeks after HepG2 cell liver injection, bioluminescence imaging (BLI) was used to estimate tumor establishment. αGPC3 was conjugated with DFO and labeled with ^89^Zr [10]. (For simplicity, ^89^Zr-DFO-αGPC3 is written as ^89^Zr-αGPC3.) Mice (n = 11 per group) were injected retro-orbitally with 8.1 to 10 megabecquerels (MBq) of ^89^Zr-αGPC3_H_ or ^89^Zr-αGPC3_M_. Mice with tumors predicted using BLI (n = 6 per group) underwent PET/CT five days after ^89^Zr-αGPC3 injection. Maximum activity concentration (MBq/mL) was measured in a 2D region of interest (ROI) to calculate tumor radioisotope uptake (percent injected dose per milliliter, %ID/mL), tumor-to-liver ratio, and tumor maximum standardized uptake value (SUV_max_). Biodistribution studies using gamma counts were performed separately in non-tumor-bearing, non-imaged mice two days after injection and in PET-imaged mice after imaging completion to determine %ID/g for select organs and tumors. Livers from PET-imaged mice were processed for histopathology. Details provided in Supplementary Methods.

## Results

### Humanized aGPC3 and αGPC3-DFO maintains GPC3 binding in vitro

**Figure 1:**
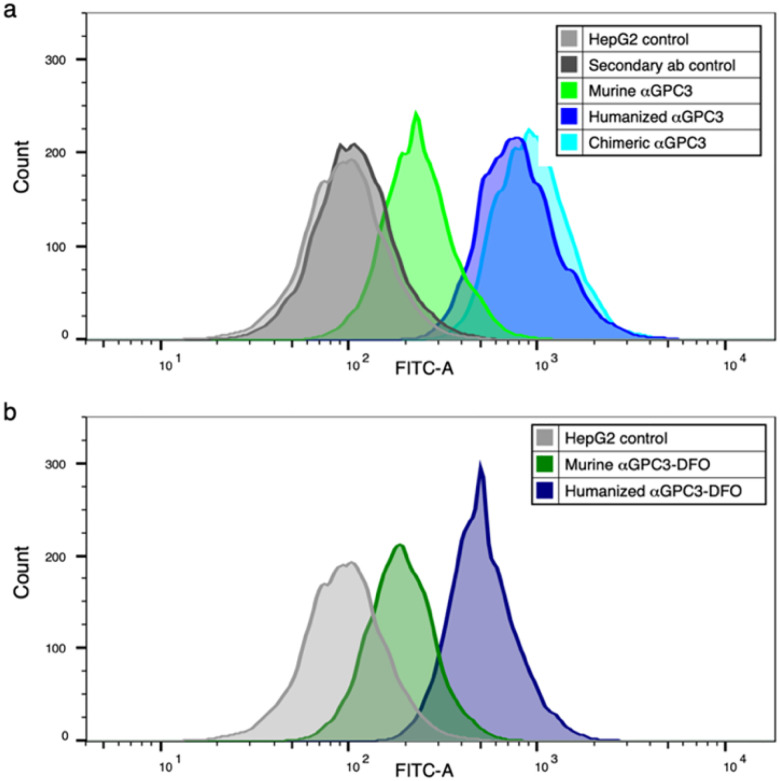
Humanized αGPC3 binds to GPC3-expressing HepG2 cells *in vitro*. (a) Flow cytometric mean fluorescence intensity (MFI) of αGPC3_M_ (green), αGPC3_C_ (aqua), and αGPC3_H_ (blue) compared with controls (unlabeled HepG2 cells and FITC-labeled secondary antibody alone, gray). (b) MFI of deferoxamine (DFO)-conjugated αGPC3_M_ (dark green) and αGPC3_H_ (navy) compared with control (gray)

Binding to HepG2 cell surface GPC3 by unconjugated αGPC3_M_, αGPC3_C_, and αGPC3_H_ was confirmed by flow cytometry (Fig. 1a). Binding of DFO-conjugated and αGPC3_M_ to GPC3 was overall similar to the unconjugated antibody (Fig. 1b). Binding of αGPC3_H_ and αGPC3_C_ to GPC3 was greater than αGPC3_M_.

### Bioluminescence imaging predicts tumor establishment

**Figure 2:**
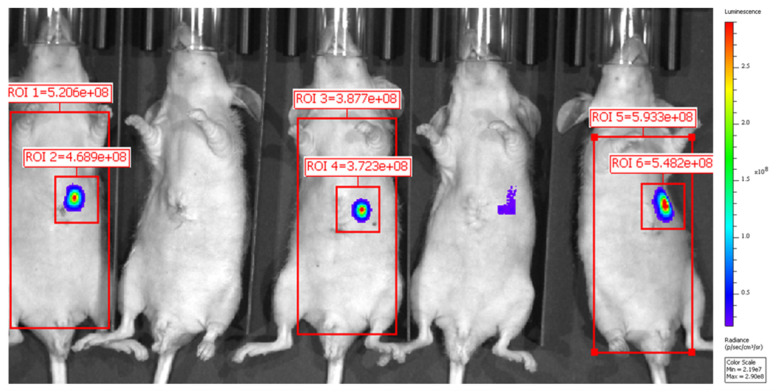
Bioluminescence imaging predicts tumor establishment. Representative BLI image demonstrating predicted tumor establishment in three of the five mice shown. ROI = region of interest. (Position 1 to 5 from left to right; mean photon emission not calculated for positions two and four)

Tumors were identified with BLI (Fig. 2). Mice were assigned to ^89^Zr-αGPC3_H_ and ^89^Zr-αGPC3_M_ injection such that mean photon emission (photons/sec) in tumor-containing ROIs was similar between groups (Table 1).

### αGPC3_H_ is amenable to ^89^Zr radiolabeling

The radiochemical purity of both ^89^Zr-αGPC3 antibodies was > 98% and the specific activity was 0.14 GBq/mg. Details provided in Supplementary Methods.

### Humanized ^89^Zr-aGPC3 immunoPET reliably identifies tumors

**Figure 3:**
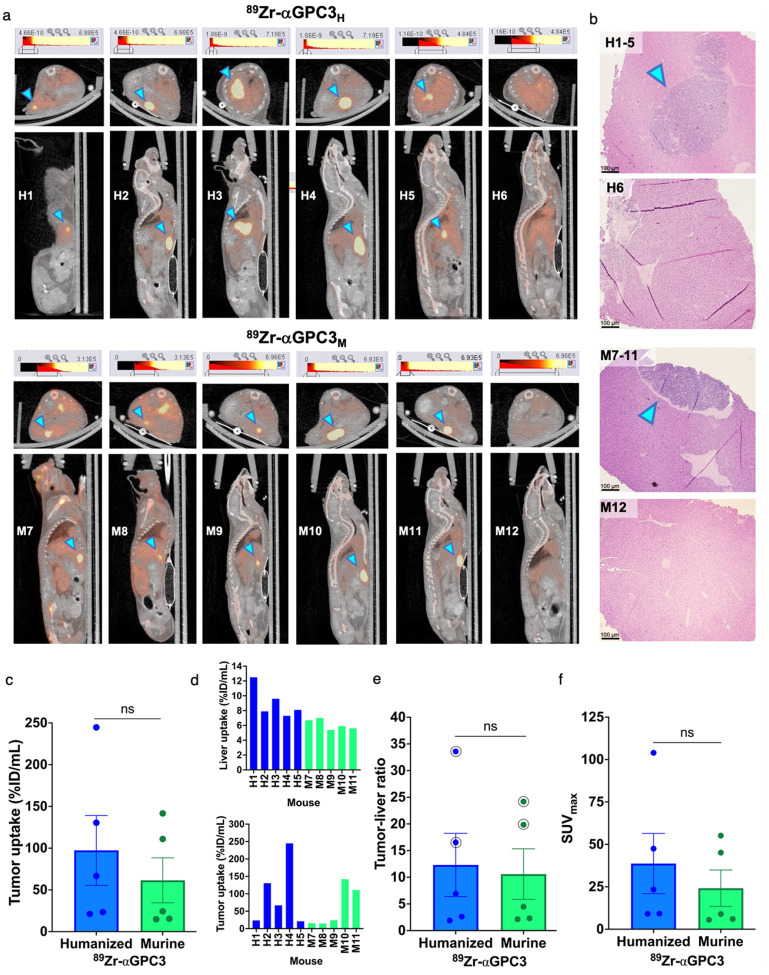
Humanized ^89^Zr-αGPC3 immunoPET reliably identifies tumors. (a) Axial (top) and sagittal (bottom) PET/CT images of ^89^Zr-αGPC3_H_- and ^89^Zr-αGPC3_M_-injected mice. (b) Select H&E-stained liver sections. Blue arrowheads denote tumors. Scale bars 100 μm. (c) Tumor radioisotope uptake (%ID/mL). (d) Liver and tumor %ID/mL by mouse. (e) Tumor-to-liver ratio of %ID/mL; circle denotes largest tumors. (f) Tumor maximum standardized uptake (SUV_max_). Each point or thin bar (d) denotes the value for each tumor-bearing mouse (n=5/group); large bars denote the mean with SEM

Five of six mice injected with ^89^Zr-αGPC3_H_ and ^89^Zr-αGPC3_M_, respectively, demonstrated discrete hepatic localizations of increased PET intensity consistent with tumors (H1–H5, M7–M11; Fig. 3a). Mean bioluminescence of PET-identified tumors (Table 1) was equivalent between groups (4.8×10^8^ vs 6.3×10^8^ +/− 4.5×10^8^ photon/sec, p = 0.75). Histopathology identified tumors in H1–H5 and M7–M11, but not in mice without PET-identified tumors (H6, M12) (Fig. 3b). Tumor uptake (97 vs 61 +/− 50%ID/mL, p = 0.42) and tumor-to-liver ratio (12 vs 11+/− 7.6, p = 0.68) were not significantly different between groups, despite significantly increased liver uptake in the ^89^Zr-αGPC3_H_-injected mice (9.1 vs 6.1 +/− 1.0%ID/mL, p = 0.02) (Fig. 3c–e). SUV_max_ was equivalent between groups (39 vs 24 +/− 21, p = 0.51) (Fig. 3f).

### No difference in tumor radioimmunoconjugate uptake on biodistribution studies

**Figure 4:**
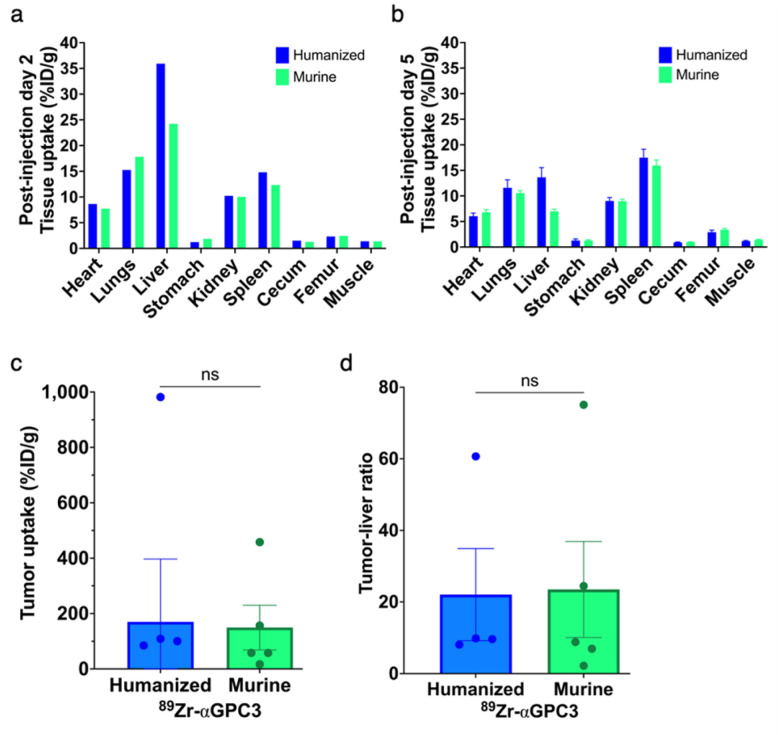
Tissue biodistribution is not significantly different between humanized and murine ^89^Zr-αGPC3-injected mice. (a-b) Mean tissue radioisotope uptake (%ID/g) in mice not imaged with PET (non-tumor-bearing) (n=5/group) (a) and imaged with PET (majority tumor-bearing) (n=6/group) (b), two and five days after injection, respectively. (c) Tumor uptake and (d) tumor-to-liver ratio (%ID/g) in PET-imaged mice. Each point denotes the value for each tumor-bearing mouse (humanized n=4 with negative value excluded (see Supplementary Methods), murine n=5); bars denote mean with SEM when applicable

In non-imaged mice, the liver had the highest %ID/g calculated from gamma counter measurements followed by the lungs and spleen, with no significant difference between ^89^Zr-αGPC3_H_ and ^89^Zr-αGPC3_M_-injected mice (mean 10 vs 8.8 +/− 4.6%ID/g, p = 0.77) (Fig. 4a). In PET-imaged mice, tumor uptake was 7-fold greater than other organs, with equivalent organ uptake (mean 7.1 vs 6.3 +/− 2.7%ID/g, p = 0.75), tumor uptake (170 vs 149 +/− 241%ID/g, p = 0.93), and tumor-to-liver ratio of %ID/g (22 vs 24 +/− 19, p = 0.94) (Fig. 4b–d).

## Discussion

Humanized αGPC3 specifically targeted GPC3 *in vitro* and *in vivo*, enabling HCC detection with immunoPET in an orthotopic xenograft mouse model. This proof-of-concept study builds on our prior research validating a murine radioimmunoconjugate for a theranostic approach to HCC, with potential to improve diagnosis, treatment, and survival [6, 10–12].

Our results demonstrate that humanization of ^89^Zr-αGPC3 did not alter the highly avid binding to GPC3 on HepG2 cells and liver tumor xenografts. First, flow cytometry established at least equivalent, if not greater, binding of αGPC3_H_ to GPC3 compared with αGPC3_M_, with minimal change when conjugated with DFO. Next, quality assurance of ^89^Zr labeling confirmed that ^89^Zr-αGPC3_H_ maintained high purity and specific activity. The majority of our experiments focused on the novel *in vivo* comparison between ^89^Zr-αGPC3_H_ and ^89^Zr-αGPC3_M_. Five of six tumors in each group were detected by immunoPET, with no difference between groups in mean IVIS bioluminescence. PET/CT data revealed no significant difference in mean tumor uptake and tumor-to-liver ratios (%ID/mL). Similarly, biodistribution analysis showed no difference in mean organ uptake, tumor uptake, and tumor-to-liver ratios (%ID/g).

While finding comparability between ^89^Zr-αGPC3_H_ and ^89^Zr-αGPC3_M_ achieved the study’s primary goal, additional details are worth noting. First, tumor uptake varied based on tumor size, with higher uptake in larger tumors as previously demonstrated [6]. %ID/g (gamma counter) results were greater than %ID/mL (PET) due to limited PET/CT spatial resolution causing partial volume effect; hence, there could be a larger discrepancy between %ID/g and %ID/mL values in mice with smaller tumors (e.g., H3, M9) (Fig. 3, Table 1). While further consideration of the clinical impact of partial volume effect is warranted, this finding does highlight the successful detection of small tumors with ^89^Zr-αGPC3 immunoPET. Second, background liver uptake was greater in the ^89^Zr-αGPC3_H_ group, which could imply Fc-mediated liver uptake of ^89^Zr-αGPC3_H_. However, our prior studies of mice injected with ^89^Zr-αGPC3_M_ compared with non-GPC3-targeting and GPC3-blocked controls demonstrated similar background liver uptake [6, 11, 16]. Furthermore, the tumor-to-liver ratio by nature adjusts for such variables, with no difference between groups suggesting that tumor uptake was also proportionally higher in the humanized antibody group. In fact, tumor-to-liver ratios of 12 or greater indicate ^89^Zr-αGPC3_H_ is highly specific for GPC3-expressing tumors [16].

Tumor presence was histopathologically confirmed in mice with PET-identified tumors, while no tumors were found on histologic analysis of livers without PET-identified tumors. A limitation here is that, while meticulous gross examination of the liver and histopathologic analysis of suspected tumors was performed, serial sectioning of the entire left hepatic lobe was not undertaken due to limited funding. Therefore, the discordance between BLI and PET for H6 and M12 is unresolved. Of note, the three-week interval between imaging modalities was longer than in previous studies and thus tumor involution may have occurred.

Our study is similar to those from other groups in that it underscores the potential of human αGPC3 to detect HCC with immunoPET, however, there are key differences. Tumor-to-liver ratios by PET/CT and biodistribution analyses were notably higher than those reported by Natarajan *et al*. using a similar ^89^Zr-labeled human αGPC3 IgG antibody and Fayn *et al*. using ^89^Zr-labeled GPC3-targeting HN3 single-domain antibodies. In addition, there was a greater relative difference between tumor uptake and uptake in organs such as the heart, lungs, gastrointestinal tract, and kidneys on biodistribution analysis [16, 17]. While different methods for model development and radioimmunoconjugate injection used may affect the results such that they are not directly comparable [16, 17], it is possible that our humanized antibody has a higher specificity for GPC3-expressing tumors. Furthermore, it should be noted that tumor-to-liver ratios were measured five days after injection in this study compared with one to seven days after injection in the aforementioned studies, however our prior experiments with ^89^Zr-αGPC3_M_ demonstrated high tumor-to-liver ratios calculated from four hours up to seven days after injection [6, 11]. Finally, Carrasquillo *et al*. conducted a phase I clinical study of PET/CT in HCC patients using αGPC3 codrituzumab labeled with iodine-124 (^124^I). While this valuable work underscores the clinical translatability of radiolabeled antibodies against GPC3, there was no tumor uptake in one patient and low tumor-to-liver ratios in several others [18]. The authors stated that ^89^Zr could have been a reasonable alternative to ^124^I, and our findings support further investigation of ^89^Zr-αGPC3 immunoPET to overcome challenges encountered with other radioimmunoconjugates. We appreciate the rigorous and ongoing work by our colleagues in the field and believe that parallel approaches to developing GPC3-targeted radiolabeled imaging agents will be beneficial [14–20, 23, 24].

In conclusion, humanized αGPC3 successfully targeted GPC3 *in vitro* and *in vivo*. Compared with our previously validated murine antibody, ^89^Zr-αGPC3_H_ immunoPET demonstrated comparable HCC detection with highly specific tumor uptake in an orthotopic xenograft mouse model, affirming the efficacy and clinical translatability of ^89^Zr-αGPC3_H_ immunoPET for HCC detection [16]. Given our GPC3-targeted murine radioimmunoconjugates were previously validated for both immunoPET and cytotoxic RIT, immediate next steps include assessing treatment response using αGPC3_H_-based RIT. This developing theranostic joins a growing field of other solid tumors, including colorectal, breast, prostate, renal cell cancers, non-Hodgkin’s lymphoma, and neuroendocrine tumors, and has the potential to transform HCC management [10, 25, 26].

## Figures and Tables

**Table 1:** Mouse-specific immunoPET and biodistribution data correlated with tumor size

	H1	H2	H3	H4	H5	H6^b^	M7	M8	M9	M10	M11	M12^b^
Biolum. (Photons/Sec)	3.7×10^8^	5.5×10^8^	^a^	9.2×10^8^	7×10^7^	1.6×10^8^	1.4×10^8^	4.1×10^8^	2.5×10^8^	2.1×10^9^	4.7×10^8^	2×10^7^
Rank^c^	5	3		2	9	7	8	6	10	1	4	11
Tumor Weight (g)	0.003	0.031	0.005	0.115	0.003		0.003	0.003	0.003	0.036	0.043	
Rank	6–10	4	5	1	6–10		6–10	6–10	6–10	3	2	
Tumor %ID/mL (PET)	24	131	67	245	21		15.5	14.9	24	142	111	
Rank	6	3	5	1	8		9	10	6	2	4	
Liver %ID/mL (PET)	12.5	7.9	9.6	7.3	8.1	8.9	6.7	7.0	5.4	5.9	5.6	4.7
Tumor: Liver	1.9	16.5	6.9	34	2.6		2.3	2.1	4.5	24	19.9	
Rank	10	4	5	1	7		8	9	6	2	3	
SUV_max_	9.2	47	23	104	9.1		6.0	5.6	9.0	55	45	
Tumor %ID/g (Gamma counter)	−426^d^	100	982	109	85		16.8	58	458	156	56	
Rank	10	5	1	4	6		9	7	2	3	8	
Liver %ID/g	20	10.2	16.2	11.3	10.5	10.9	7.6	8.3	6.1	6.4	6.5	7.7
(Gamma counter)												
Tumor: Liver	−21^d^	9.8	61	9.6	8.1		2.2	7.0	75	24	8.8	
Rank	10	4	2	5	7		9	8	1	3	6	

a Not measured due to minimal apparent signal.

b H6, M12: no tumor identified on PET/CT or histopathology (blank = not applicable).

c Rank from largest (1) to smallest (10/11).

d −426 included in Fig. 4c calculations; −21 excluded from Fig. 4d (see Supplementary Methods).

## Data Availability

All data will be available after publication upon request to the corresponding author.

## Abbreviations

HCC: hepatocellular carcinoma
CT: computed tomography
immunoPET: immuno-positron emission tomography
RIT: radioimmunotherapy
GPC3: glypican-3
^89^Zr: zirconium-89
αGPC3_M_: murine antibody targeting GPC3
αGPC3_H_: humanized αGPC3
αGPC3_C_: chimeric intermediary
DFO: deferoxamine
ROI: region of interes
SUV_max_: maximum standardized uptake value
^124^I: iodine-124
